# Delivering a Home-Based Exercise Program to Youth With Osteogenesis Imperfecta: Protocol for a Comparative-Approach Study

**DOI:** 10.2196/40262

**Published:** 2023-07-03

**Authors:** Georgia Powell, Marianne Gagnon, Svetlana Komarova, Frank Rauch, Louis-Nicolas Veilleux

**Affiliations:** 1 Department of Surgery Division Surgical and Interventional Sciences McGill University Montreal, QC Canada; 2 Shriners Hospitals for Children-Canada Montreal, QC Canada; 3 Faculty of Dental Medicine and Oral Health Sciences, McGill University Montreal, QC Canada; 4 Department of Pediatrics, McGill University Montreal, QC Canada

**Keywords:** adherence, cost-effectiveness, exercise, osteogenesis imperfecta, telemedicine

## Abstract

**Background:**

Osteogenesis imperfecta (OI) is a rare bone fragility disorder associated with muscle weakness. Individuals with OI may therefore benefit from exercise interventions aiming to improve muscle and bone strength. Given the rarity of OI, many patients do not have access to exercise specialists who are familiar with the disorder. As such, telemedicine, the provision of health care through technology to provide care at a distance, may be well suited for this population.

**Objective:**

The main objectives are (1) to investigate the feasibility and cost-effectiveness of 2 telemedicine approaches for the delivery of an exercise intervention for youth with OI and (2) to assess the impact of the exercise intervention on muscle function and cardiopulmonary fitness in youth with OI.

**Methods:**

Patients with OI type I (the mildest form of OI; n=12, aged 12-16 years) from a pediatric orthopedic tertiary hospital will be randomized to receive a 12-week remote exercise intervention in either (1) a supervised group (n=6), monitored every session, or (2) a follow-up group (n=6), receiving monthly progress update appointments. Participants will undergo the following pre- and postintervention evaluations: sit-to-stand test, push-up test, sit-up test, single-legged balance test, and a heel-rise test. Both groups will be given the same 12-week exercise regimen, which includes cardiovascular, resistance, and flexibility training. For each exercise training session involving the supervised group, a kinesiologist will provide instructions to participants through live video sessions using a teleconferencing application. On the other hand, the follow-up group will discuss their progress with the kinesiologist every 4 weeks over a teleconferencing video call. Feasibility will be assessed by recruitment, adherence, and completion rates. A cost-effectiveness analysis of both approaches will be computed. Changes in muscle function and cardiopulmonary fitness will be examined between the 2 groups, pre- and postintervention.

**Results:**

It is anticipated that the supervised group will have higher adherence and completion rates compared to the follow-up group, which may be associated with greater physiological benefits; however, it may not be as cost-effective compared to the follow-up approach.

**Conclusions:**

By determining the most feasible telemedicine approach, this study may serve as a basis for providing increased access to specialized adjunct therapies for individuals with rare disorders.

**International Registered Report Identifier (IRRID):**

PRR1-10.2196/40262

## Introduction

Osteogenesis imperfecta (OI) is a rare congenital disorder characterized by bone fragility occurring in approximately 1 in every 10,000 individuals [1]. OI is typically caused by a dominant mutation in 1 of the 2 genes that code for the alpha chains in collagen type I*, COL1A1* and *COL1A2* [1]*.* The Sillence clinical classification of OI is divided into 4 phenotypic groups, with type I being the mildest and most frequently diagnosed [1,2]. OI type I comprises individuals with bone fragility and those whose stature falls within or close to normal limits [2,3]. While bone fragility is the leading characteristic of OI, we have shown that children and adolescents with OI type I have lower peak muscle force and muscle size compared to age- and sex-matched controls [4,5]. Given the positive relationship between muscle force and bone strength, muscle function deficits are likely to contribute to bone mass deficits in youth with OI [4-6].

For both healthy children and children with OI type I, exercise has been shown to be an effective method for improving cardiopulmonary fitness, as well as muscle strength [7,8]. Therefore, given the previously mentioned muscle deficits in children and adolescents with OI [4,5], individuals with OI type I may be more likely to benefit to a greater extent from this adjunct therapy, in addition to the typical mainstay of treatment, which includes rehabilitation programs, bisphosphonate therapy, and orthopedic surgery [9]. However, given the rarity of the disorder, many patients have challenges accessing exercise specialists who are familiar with the treatment of OI; thus, patients often seek or receive care from specialized tertiary hospitals. Attending both regular exercise training interventions in addition to frequent therapeutic visits at tertiary care centers may present a barrier for many patients, as specialized hospitals are often located in large urban areas, which can be far from patients’ homes.

Telemedicine is a method for the remote delivery of professional health services at a distance by linking clinicians and patients through information technology [10,11]. Lambert et al [12] used a telemedicine approach for the delivery of a 16-week home exercise program for pediatric acute lymphoblastic leukemia survivors. This supervised telemedicine approach was shown to be feasible, as the results demonstrated a mean adherence rate of 89% and a completion rate of 75% [12]. In contrast, Gagnon et al [13] used a follow-up telemedicine approach for the delivery of a 12-week home exercise program for youth with arthrogryposis multiplex congenita, where clinicians conducted follow-ups with study participants every 3 weeks. The results showed a 68% adherence rate and a 64% completion rate, which was sufficient for most study participants to achieve their goals [13]. As the recent literature demonstrates, the follow-up approach may be less effective as it led to a lower completion rate [12,13]. However, additional clinician time cost associated with the supervised approach may exceed the increase in benefits associated with constant supervision.

There remains a gap in the literature with respect to the comparison between supervised and follow-up telemedicine approaches and the associated cost-effectiveness of both approaches. Hence, the aim of this study is to evaluate the feasibility and cost-effectiveness of 2 telemedicine approaches (supervised vs follow-up) for the delivery of a home exercise program. The secondary aim of the study is to investigate the effectiveness of the home-based exercise program on muscle function and cardiopulmonary fitness in youth with OI type I. Given the aims of this study, it is hypothesized that participants in the supervised group will have higher adherence and completion rates compared to the follow-up group, which will be further associated with greater increases in muscle strength and cardiopulmonary fitness from the exercise intervention. However, the additional intervention delivery time required for the supervised group may make it less cost-effective compared to the follow-up approach.

## Methods

### Study Design

This pilot study examines the feasibility of delivering an exercise intervention to a pediatric and youth population with OI by means of telemedicine. Study participants will be randomized into 1 of 2 telemedicine approach groups: (1) the supervised group and (2) the follow-up group. Given the COVID-19 pandemic, this protocol was designed to be conducted remotely, including the assessments. Therefore, simple clinical tests rather than hospital-based evaluations, which require cutting-edge technology, were favored.

### Participant Eligibility

Individuals with a diagnosis of OI type I based on clinical evaluation and genetic mutation who are followed at a tertiary pediatric orthopedic hospital, ranging in age from 12 to 16 years, will be invited to participate in this study. Inclusion criteria encompass (1) confirmed genetic mutation in *COL1A1* or *COL1A2* genes; (2) medical approbation to initiate an exercise program by the patient’s treating physician; (3) the ability to communicate in either English or French; and (4) the availability of the internet, a webcam, and a microphone. The exclusion criteria include (1) fractures of long bones in the lower or upper limbs within the past 12 months, (2) fractures of the spine within the past 12 months, (3) lower and upper limb long bone or spinal surgery within the past 12 months, and (4) location outside of the province of Quebec due to professional license restrictions.

### Ethical Considerations

Study approval was granted by McGill University’s Faculty of Medicine and Health Sciences Institutional Review Board (A01-M01-17A). Informed consent forms will be provided for participants aged 14 years and older. Any participant younger than 14 years will be asked to sign an assent form. Additionally, for those younger than 14 years, parents or caregivers will be required to sign a parental consent form as per Quebec’s provincial regulations. Individuals who meet the study inclusion criteria and agree to participate in the research study will be deidentified and assigned a study ID number. Direct subject identifiers will be stored as key codes and kept separate from the study data set. Patient’s medical record number, date of birth, and corresponding study ID number will be saved in a password-protected file that will be stored on a hospital computer in the office of the principal investigator. The key code will be the only link that reidentifies the study participant to their health information; all other patient data will be deidentified. All study records will be retained and stored in the principal investigator’s office for 7 years. Study participants will not receive compensation for their participation.

### Recruitment

A total of 12 patients with OI type I will be recruited at a tertiary orthopedic hospital. Given that this study will be conducted remotely, patients who meet the study eligibility criteria will be contacted by the study coordinator over the phone. Those who are interested in participating will receive a follow-up email from the study coordinator with the appropriate consent or assent form, providing them with more information regarding their participation. Based on participant’s preference, consent or assent forms will be either emailed or mailed to their place of residence. To ensure privacy, all emails from the study coordinator will be sent encrypted. Study participants will be randomized into either (1) the supervised group or (2) the follow-up group. Study personnel will produce a block randomization list using a random number generating software, which will be sent to the principal investigator. Each randomized number will be written on piece of paper and placed in an envelope. Once a study participant is enrolled, a hospital employee who is not involved in the study will select one of the envelopes and deliver it to the study personnel, who will assign the study participant to the specified group.

### Evaluations and Intervention

#### Overview

The duration of the study is 16 weeks: 12 weeks for the intervention and 2 weeks at the beginning and end for evaluations. All participants will be given an exercise program to be completed at home. Both groups will use the teleconferencing application Microsoft Teams (version 1.3.00.28, Microsoft Corp). The supervised group will be monitored every session, while the follow-up group will receive feedback every 4 weeks.

**Figure 1:**
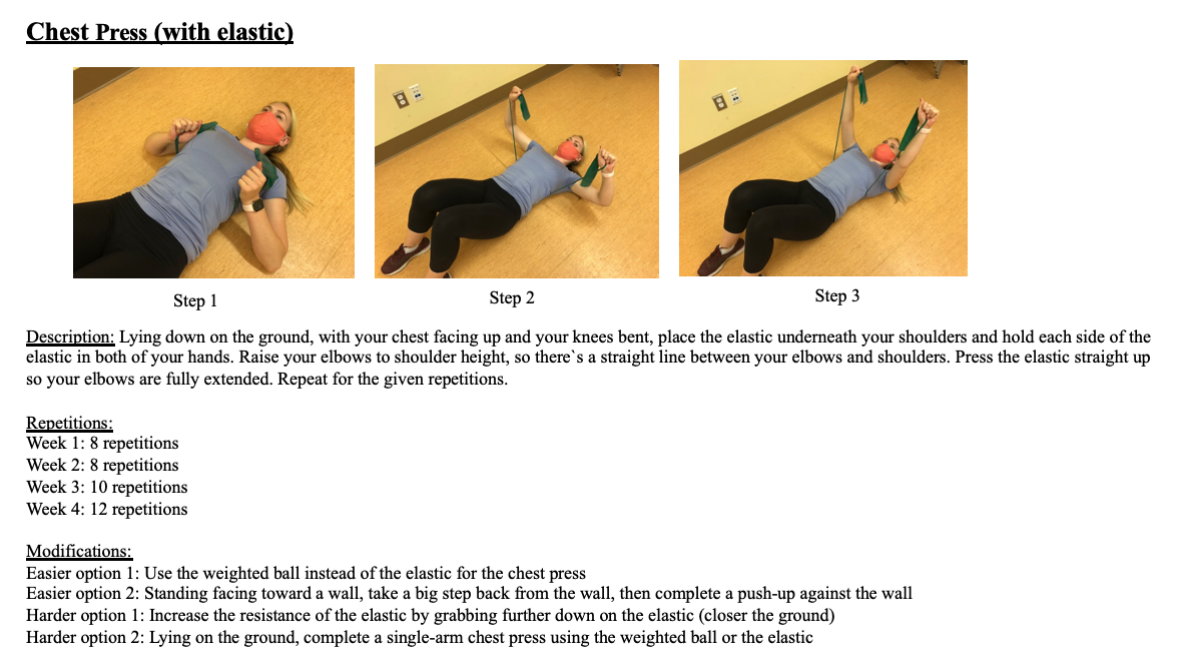
Home-exercise program instructions.

Previous to the intervention, participants will be provided with initial testing and tutorials on the telemedicine application (Microsoft Teams) and the activity and heart rate monitor (Polar A370 watch; Polar Electro). Participants will be mailed the required equipment, such as the Polar A370 activity monitor (Polar watch), a 5-pound exercise ball, and a resistance band. All attempts possible have been made to ensure this study uses a minimal amount of equipment in order to reach a larger population that may have limited access to resources while also ensuring no sacrifices are made to the overall quality of the study. Both video and written instructions in English and French will be available to participants throughout the study. Instructions for the following will be included: telemedicine connections, completion of questionnaires, and Polar watch details (Bluetooth and USB connections, charging, and data transfer). Additionally, all study participants will have access to the 12-week exercise plan throughout the entire study. The exercise plan includes an illustration, video, and written description of each exercise, as well as 2 easier and 2 more difficult modifications for each movement (Figure 1). The exercise plan comprises 6 warm-up exercises, 7 to 8 exercises that will be repeated 3 times, and 5 cool-down stretches.

#### Evaluations

##### Overview

To assess the impact of the 12-week exercise program, a comparison of pre- and postintervention evaluations will take place (Table 1). The pre- and postintervention evaluations will be recorded on Microsoft Teams in order to better assess and compare participants’ progress before and after the intervention. Throughout the evaluations, study personnel will ask participants to have a parent or caregiver supervising nearby. During the pre- and postintervention evaluations, participants will be asked to complete the following three Patient-Reported Outcomes Measurement Information System (PROMIS) questionnaires: (1) Mobility Short Form, (2) Pain Interference Short Form, and (3) Physical Activity Short Form. Previous research has shown that PROMIS questionnaires have been successfully validated and are clinically feasible for a pediatric population [14]. These questionnaires will establish a baseline indicator for participant’s pain, mobility, and physical activity levels previous to the intervention. Participant's pre- and postintervention questionnaires will be compared to determine what changes, if any, were realized by the participant.

**Table 1:** Pre- and postintervention evaluation tests.

Test	Assessment	Parameter
Sit-to-stand test	Cardiopulmonary fitness and lower body muscular strength	Heart rate (beats per minute) and total number of repetitions performed
Single-legged balance test	Visual and proprioceptive inputs	Time (seconds)
Push-up test	Upper body muscular endurance	Total number of repetitions performed
Sit-up test	Abdominal strength	Total number of repetitions performed
Heel-rise test	Lower body muscular endurance	Time (seconds)

##### Sit-to-Stand Test

The sit-to-stand test assesses cardiopulmonary fitness and lower body muscle strength [15,16]. The participant will sit down and stand up from a chair with their hands on their hips as many times as possible within 1 minute. The participant’s heart rate (in beats per minute) will be taken immediately before, during, and after the test, which will be measured on the Polar A370 heart rate monitor. The number of repetitions completed will be recorded at the end of the test and expressed in absolute value [16]. Additionally, the number of completed repetitions will be expressed as the product of body weight to provide an index of total work (repetitions × weight in kg) [16,17].

##### Single-Legged Balance Test

Postural control will be assessed with 2 visual conditions: eyes open and eyes closed. The participant will stand on 1 leg with their arms crossed over their chest for up to 45 seconds. The participant will complete both visual conditions on each foot. Throughout the test, study personnel will ask the participant’s parents or caregivers to stand close by for support in case the participant loses their balance. The duration of time the participant can maintain their balance under each condition will be collected [7].

##### Push-Up Test

The push-up test assesses upper-body muscular endurance of the chest, shoulders, and arms. The participant will perform as many consecutive push-ups as they can with no time limit. The test will be stopped if the participant is unable to maintain proper technique over 2 consecutive repetitions. Following the Canadian Society of Exercise Physiology, Physical Activity Training for Health (CSEP-PATH) guidelines, female participants will use their knees as their pivot point, whereas male participants will use their toes as their pivot point [7].

##### Sit-Up Test

The sit-up test will evaluate abdominal strength. The participant will complete as many sit-ups as they can within 1 minute. The participant will rise to a seated position from lying down with their knees bent and their arms crossed over their chest [7].

##### Heel-Rise Test

The heel-rise test evaluates muscular endurance of the lower limbs [18]. The participant will complete 2 trials of raising their heels 20 times in a row, as fast as possible, with their arms crossed over their chest. Participants will be given a rest period of 2 minutes between the 2 trials. Ankle plantar flexor strength is scored from 0 to 5, based on the number of heel rises the participant is able to complete [18].

#### Exercise Intervention

**Figure 2:**
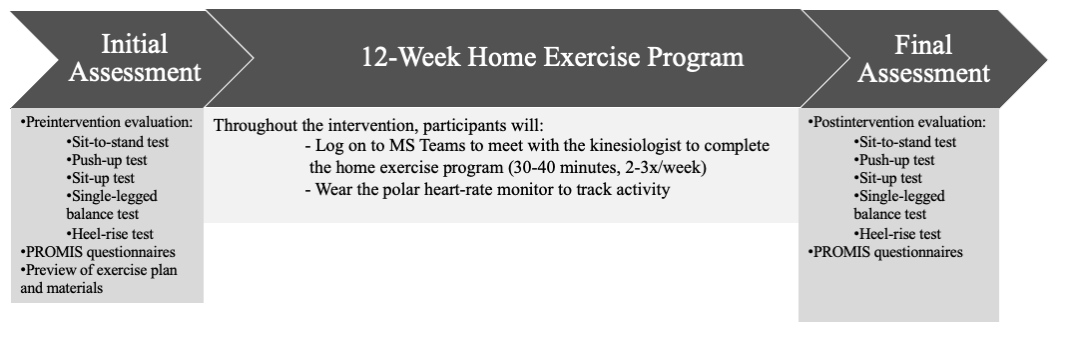
Summary of the 12-week home exercise program for the supervised group. PROMIS: Patient-Reported Outcomes Measurement Information System.

**Figure 3:**
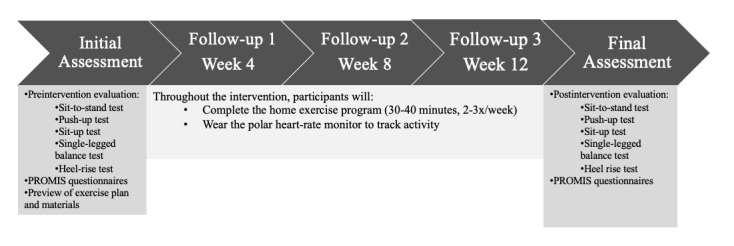
Summary of the 12-week home exercise program for the follow-up group.

The exercise intervention will begin with 35 minutes twice a week, including a warmup and cooldown, each 5 minutes in duration. Every 4 weeks, the training will intensify in either duration of exercise or number of sessions per week, which is required to stimulate physiological adaptation and enhance improvement [7]. For the duration of the intervention, a kinesiologist (human movement specialist) will provide training and supervision to participants in the supervised group at home through live video sessions. Participants in the supervised group will be separated into 2 groups of 3 participants. For the follow-up group, the kinesiologist will meet with participants individually every 4 weeks to review their progress, preview the next month’s exercise plan, and address any concerns or challenges the participants may have. The exercise program timelines for both groups are outlined below (Figures 2 and 3).

### Measurement

#### Feasibility

In determining the feasibility of the telemedicine approaches for the delivery of an exercise intervention, recruitment, adherence, and completion rates will be measured. Recruitment rate will be calculated by dividing the study sample by the total number of contacted potential participants [19]. Adherence rate is defined as the percentage of the exercise intervention sessions completed per participant [20]. To monitor participants’ adherence to the exercise intervention, data exported from the activity monitor (Polar watch) will be recorded by study personnel on an activity log for all participants in both groups. Finally, completion rate is defined as the number of participants that completed the exercise intervention as a percentage of the overall number of participants that began the intervention [20].

#### Cost

To determine the cost comparison between the 2 groups, the number of hours spent by the kinesiologist delivering the exercise intervention to the supervised group will be recorded and compared to the number of hours spent with participants in the follow-up group. The kinesiologist will be paid a rate which is commensurate with their previous experience as well as a wage that aligns with the rate of pay for a kinesiologist in the market in which this study is conducted. The equipment costs (Microsoft Teams, Polar heart rate monitor, resistance band, and weighted exercise ball) will be reported and included in the calculations. When drawing cost comparisons for the 2 approaches, the supervised approach is a group approach, whereas the follow-up approach is an individualized approach. Therefore, a direct comparison between the 2 approaches presents a challenge. Nevertheless, to mitigate the difference in group and individualized approaches, the cost comparison for both the supervised and follow-up groups will be computed and presented as a cost per participant.

#### Cost-Effectiveness

To compare the cost-effectiveness of the 2 telemedicine approaches, a bottom-up approach will be used, a method for the assessment of each resource required to provide health care and assigning the related costs accordingly [21,22]. For both the supervised and follow-up groups, the mean performance outcome for each of the 5 preintervention evaluation tests will be computed. These means are then compared within their group (supervised or follow-up) to a mean for the same 5 postintervention evaluations, determining the mean outcome (increase or decrease) for each of the 5 tests. These mean outcomes will then be compared to the total cost of each telemedicine approach to determine the cost (on a per-unit basis) of the health outcome. Doing so allows for the assessment of not only which telemedicine approach has a higher cost overall, but most importantly, the per-unit cost of the health outcome for the supervised and follow-up groups. This provides a more accurate comparison, with respect to financial costs, of the 2 methods.

### Statistical Analysis

As this is a feasibility study, the testing procedure will be assessed rather than only focusing on the potential outcomes of the intervention. To determine the optimal clinical practice of telemedicine for an exercise intervention, recruitment, adherence, and completion rates of 2 randomized groups (supervised and follow-up approaches) will be investigated. Descriptive statistics will be presented as means and SDs. Based on previous research on home-based exercise interventions [23], the adherence rate will be assessed with a set threshold of 80%. A Wilcoxon rank sum test will be used to evaluate the adherence rate and compare both telemedicine approaches. Whereas both recruitment and completion rates will be compared using descriptive statistics and assessed with a set threshold of 50%. To measure the change in outcome parameters of participants’ muscle strength and cardiopulmonary fitness, pre- and postintervention evaluation results will be assessed with a Friedman ANOVA. A Kruskal-Wallis test will be used to determine the differences in outcome parameter changes between the 2 groups (supervised and follow-up groups).

To assess the cost-effectiveness of the 2 telemedicine approaches, a cost-effectiveness plane and an incremental cost-effectiveness ratio will be computed. The incremental cost and incremental effect will be plotted on a cost-effectiveness plane, where the horizontal axis will represent the incremental cost and the vertical axis will represent the incremental effect [24,25]. The costs and clinical outcomes for both telemedicine groups will be plotted on the plane, which will visually demonstrate the additional cost per unit of health gain for each telemedicine approach. To calculate the incremental cost-effectiveness ratio, the difference in costs will be divided by the difference in mean performance outcomes between the supervised and follow-up groups.

Finally, as OI is classified as a rare disorder [1], analyses will be carried out with a significance level (α) of 0.10 [26]. Statistical analyses will be conducted using the statistical analysis software SPSS, version 25 (SPSS Inc).

## Results

As the costs associated with both telemedicine approaches can be estimated, the anticipated cost comparison and breakdown of each approach are summarized below (Table 2 and Textbox 1).

**Table 2:** Estimated cost comparison per telemedicine approach.

Cost estimations		Supervised group, CAD $ (US $)	Follow-up group, CAD $ (US $)
**Kinesiologist**			
	Per group	2700 (2006)	1620 (1204)
	Per participant	450 (334)	270 (201)
**Equipment**			
	Per group	1646 (1223)	1646 (1223)
	Per participant	274 (204)	274 (204)
**Microsoft Teams**			
	Per group	74 (55)	74 (55)
	Per participant	12 (9)	12 (9)
Total group cost estimation		4420 (3284)	3340 (2482)
Total per participant cost estimation		736 (547)	556 (414)

**Estimated cost comparison breakdown per telemedicine approach.**

**Kinesiologist**

- Evaluations
  2 hours (preintervention evaluation)+2 hours (postintervention evaluation)×6 participants=24 hours
- Intervention
  Estimated time required for the supervised approach (8 hours [month 1]+12 hours [month 2]+18 hours [month 3])×2 (number of groups)=76 hours 24 hours (evaluations)+76 hours (intervention)=100 hours Total: 100 hours (total hours)×CAD $27 (US $20) (hourly rate)=CAD $2700 (US $2006)
  Estimated time required for the follow-up approach (2 hours [month 1]+2 hours [month 2]+2 hours [month 3])×6 (number of participants)=36 hours 24 hours (evaluations)+36 hours (intervention)=60 hours Total: 60 hours (total hours)×CAD $27 (US $20) (hourly rate)=CAD $1620 (US $1204)

**Equipment**

- Per participant: CAD $8.30 (US $6.17) (resistance band)+CAD $258.75 (US $192.26) (polar watch)+CAD $7.23 (US $5.37) (weighted ball)=CAD $274.28 (US $203.80)
- Per approach: CAD $274.28 (US $203.80) ×6 (number of participants)=CAD $1646 (US $1223)

**Microsoft Teams**

- Per approach: CAD $18.40 (US $13.67) (monthly subscription fee)×4 (number of months)=CAD $74 (US $55)

## Discussion

### Overview

The aim of this study is to evaluate the feasibility of 2 telemedicine approaches for the delivery of a home-based exercise program and determine the most cost-effective approach. The secondary aim of the study is to investigate the effect of a home-based exercise program on muscle function and cardiopulmonary fitness in youth with OI type I. Given the aims of the study, it is hypothesized that the supervised group will have higher adherence and completion rates compared to the follow-up group, and therefore, the physiological benefits will be greater in the supervised group than the follow-up group due to additional benefits from constant supervision. However, the follow-up group will receive individual attention in contrast to the supervised group, in which the specialist will work with groups of 3 patients. Direct comparison of patient performance in the supervised and follow-up groups will allow us to identify which telemedicine approach is more cost-effective and efficient for the delivery of an exercise program to OI pediatric patients.

Greater adherence rates can be associated with benefits such as increased positive reinforcement, improved exercise techniques, and higher self-efficacy [12]. Furthermore, direct supervision is associated with a greater overall volume of exercise achieved and potentially more efficient training, as with this approach, clinicians have the ability to adjust exercises in real time to prevent injury or pain [12]. However, in the follow-up approach, there is schedule flexibility, as participants can perform the intervention sessions when it’s most convenient to them. As well, participants may develop autonomy by completing these sessions independently, which in the long term may lead to long-lasting physical activity as individuals feel more confident in their abilities to exercise on their own. Whereas participants in the supervised group rely on study personnel for instruction and motivation and may not develop the same level of autonomy as individuals in the follow-up group. Although there are several health benefits associated with direct supervision, these advantages may not outweigh the increased cost associated with this approach. Thus, the insight gained from this study into the level of participant supervision necessary for a telemedicine approach to be effective will satisfy the current gap in literature. The results pertaining to the feasibility and cost-effectiveness of both the supervised and follow-up telemedicine approaches will provide clinicians with fundamental knowledge regarding which approach is most feasible for the remote delivery of care to a pediatric and youth population.

To date, little is known about the potential effectiveness of exercise for children and adolescents with OI. Previous research has shown that children and adolescents with OI type I experience muscle weakness in addition to bone fragility [4]. Consequently, this muscle weakness may contribute to bone fragility elicited by the genetic mutation in the *COL1A1* or *COL1A2* genes [5]. Given the severe bone fragility in patients with OI and the strong positive relationship between muscle force and bone strength, therapies aiming to improve muscle force may lead to an increase in bone strength [5]. The results of this study will shed light on the physiological response to exercise in children and adolescents with OI. Takken et al [27] evaluated cardiopulmonary fitness and muscle strength in children and adolescents with OI type I and determined that cardiopulmonary exercise tolerance and muscle strength were significantly reduced in patients with OI. To the best of our knowledge, Van Brussel et al [8] is the only published study which demonstrated significant improvements in both aerobic capacity and muscle force and reduced fatigue levels in children and adolescents with OI type I and type IV following a 12-week supervised exercise program. On the contrary, from 2 different OI mice models, results from Tauer et al [28] and Gremminger et al [29] showed very mild improvements in muscle force and tolerance to fatigue following long-term (multiple weeks) exercise training, suggesting that in OI, there may be a decreased physiological response to exercise. Results from the proposed study will further build upon Van Brussel et al [8], providing insight into the frequency and intensity of the stimulus required to trigger physiological improvements following a 12-week exercise program for youth with OI type I.

### Limitations

The limitations of this study are the technical difficulties associated with telemedicine that may arise for both study personnel and participants, such as a poor internet connection, which may lead to low-quality, incomplete, or missed training sessions for those in the supervised group. For the follow-up group, a limitation to consider is the potential that participants will not wear the heart rate monitor during the sessions, as there will not be any direct supervision to provide reminders. This would lead to missed training data, limiting the quantity of data available for comparison between the 2 groups. Although this study was designed primarily to assess the feasibility of delivering a home-based exercise intervention through telemedicine, the data collected regarding the potential change in muscle function and cardiopulmonary fitness and the small sample size of only 12 participants may limit the ability to draw any strong conclusions. As well, given the narrow age range, the results of this study are not a predictor of younger or older patient populations; therefore, a larger-scale study would be beneficial. However, given the paucity of data regarding the physiological response to exercise in youth with OI, preliminary data will provide the basis for a larger intervention study. Additionally, as OI is primarily characterized by bone fragility, given this study’s remote delivery methods, it is not possible to assess the skeletal response to the 12-week exercise program. Thus, to build upon this study and given the skeletal deficits associated with OI, future research should investigate the impact of an exercise program on bone mineral content in individuals with OI. Recent research from Sinkam et al [30] demonstrated reduced mechanical tendon properties in a dominant severe OI mouse model. Given these results and the fact that tendons are typically rich in collagen type I, in addition to investigating skeletal parameters, researchers should evaluate the physiological response of tendons in OI from an exercise intervention.

The completion of a 12-week exercise program may present a challenge for some participants, especially during the final 4 weeks of the exercise intervention when the number of sessions increases to 3 times per week. To mitigate this potential concern, study participants in the supervised group will have the flexibility of completing missed training sessions on their own schedule and therefore have access to the exercise plan with detailed instructions, images, and videos of each exercise. Whereas participants in the follow-up group are given the flexibility of completing the training sessions when convenient to the individual rather than a rigid weekly schedule.

### Conclusion

Telemedicine provides health care professionals with the opportunity to deliver consistent care to individuals with rare disorders, such as OI, who may have previously been unable to obtain access to such treatments. Given that the COVID-19 pandemic has imposed great challenges in the delivery of health care, this study will provide beneficial insight into the feasibility and cost-effectiveness of remote health care delivery methods. If this study is successful, guidelines and recommendations may emerge, providing individuals with OI with a higher level of targeted care geared toward increasing their quality of life. Finally, the results of this study may be advantageous for the treatment of other rare disorders, furthering the possibility to provide access to care to a far greater patient population.

## Abbreviations

CSEP-PATH: Canadian Society of Exercise Physiology, Physical Activity Training for Health
OI: osteogenesis imperfecta
PROMIS: Patient-Reported Outcomes Measurement Information System

## Data Availability

Data sharing does not apply to this study as this is a protocol paper; no data sets were generated or analyzed at this point.

## References

[1] Forlino A, Marini JC (2016). Osteogenesis imperfecta. Lancet.

[2] Sillence DO, Senn A, Danks DM (1979). Genetic heterogeneity in osteogenesis imperfecta. J Med Genet.

[3] Rauch F, Glorieux FH (2004). Osteogenesis imperfecta. Lancet.

[4] Veilleux L-N, Lemay M, Pouliot-Laforte A, Cheung MS, Glorieux FH, Rauch F (2014). Muscle anatomy and dynamic muscle function in osteogenesis imperfecta type I. J Clin Endocrinol Metab.

[5] Veilleux LN, Pouliot-Laforte A, Lemay M, Cheung MS, Glorieux FH, Rauch F (2015). The functional muscle-bone unit in patients with osteogenesis imperfecta type I. Bone.

[6] Veilleux LN, Trejo P, Rauch F (2017). Muscle abnormalities in osteogenesis imperfecta. J Musculoskelet Neuronal Interact.

[7] Canadian Society for Exercise Physiology (2013). CSEP Physical Activity Training for Health.

[8] Van Brussel M, Takken T, Uiterwaal CSPM, Pruijs HJ, Van der Net J, Helders PJM, Engelbert RHH (2008). Physical training in children with osteogenesis imperfecta. J Pediatr.

[9] Trejo P, Rauch F (2016). Osteogenesis imperfecta in children and adolescents-new developments in diagnosis and treatment. Osteoporos Int.

[10] Qian W, Lam TT-N, Lam HHW, Li C-K, Cheung YT (2019). Telehealth interventions for improving self-management in patients with hemophilia: scoping review of clinical studies. J Med Internet Res.

[11] Hayden JA, van Tulder MW, Tomlinson G (2005). Systematic review: strategies for using exercise therapy to improve outcomes in chronic low back pain. Ann Intern Med.

[12] Lambert G, Alos N, Bernier P, Laverdière C, Kairy D, Drummond K, Dahan-Oliel N, Lemay M, Veilleux LN (2021). Home-based telehealth exercise intervention in early-on survivors of childhood acute lymphoblastic leukemia: feasibility study. JMIR Cancer.

[13] Gagnon M, Merlo GM, Yap R, Collins J, Elfassy C, Sawatzky B, Marsh J, Hamdy R, Veilleux LN, Dahan-Oliel N (2021). Using telerehabilitation to deliver a home exercise program to youth with arthrogryposis: single cohort pilot study. J Med Internet Res.

[14] Hinds PS, Wang J, Cheng YI, Stern E, Waldron M, Gross H, DeWalt DA, Jacobs SS (2019). PROMIS pediatric measures validated in a longitudinal study design in pediatric oncology. Pediatr Blood Cancer.

[15] Reychler G, Audag N, Mestre NM, Caty G (2019). Assessment of validity and reliability of the 1-minute sit-to-stand test to measure the heart rate response to exercise in healthy children. JAMA Pediatr.

[16] Reychler G, Pincin L, Audag N, Poncin W, Caty G (2020). One-minute sit-to-stand test as an alternative tool to assess the quadriceps muscle strength in children. Respir Med Res.

[17] Gruet M, Peyré-Tartaruga LA, Mely L, Vallier JM (2016). The 1-minute sit-to-stand test in adults with cystic fibrosis: correlations with cardiopulmonary exercise test, 6-minute walk test, and quadriceps strength. Respir Care.

[18] Caudill A, Flanagan A, Hassani S, Graf A, Bajorunaite R, Harris G, Smith P (2010). Ankle strength and functional limitations in children and adolescents with type I osteogenesis imperfecta. Pediatr Phys Ther.

[19] Kahan BC (2016). Using re-randomization to increase the recruitment rate in clinical trials - an assessment of three clinical areas. Trials.

[20] Lin E, Nguyen CH, Thomas SG (2019). Completion and adherence rates to exercise interventions in intermittent claudication: traditional exercise versus alternative exercise-a systematic review. Eur J Prev Cardiol.

[21] May AM, Bosch MJC, Velthuis MJ, van der Wall E, Steins Bisschop CN, Los M, Erdkamp F, Bloemendal HJ, de Roos MAJ, Verhaar M, Ten Bokkel Huinink DTB, Peeters PHM, de Wit GA (2017). Cost-effectiveness analysis of an 18-week exercise programme for patients with breast and colon cancer undergoing adjuvant chemotherapy: the randomised PACT study. BMJ Open.

[22] Chapko MK, Liu CF, Perkins M, Li YF, Fortney JC, Maciejewski ML (2009). Equivalence of two healthcare costing methods: bottom-up and top-down. Health Econ.

[23] Manchola-González JD, Bagur-Calafat C, Girabent-Farrés M, Serra-Grima JR, Pérez RÁ, Garnacho-Castaño MV, Badell I, Ramírez-Vélez R (2020). Effects of a home-exercise programme in childhood survivors of acute lymphoblastic leukaemia on physical fitness and physical functioning: results of a randomised clinical trial. Support Care Cancer.

[24] Fenwick E, Marshall DA, Levy AR, Nichol G (2006). Using and interpreting cost-effectiveness acceptability curves: an example using data from a trial of management strategies for atrial fibrillation. BMC Health Serv Res.

[25] Cohen DJ, Reynolds MR (2008). Interpreting the results of cost-effectiveness studies. J Am Coll Cardiol.

[26] Bogaerts J, Sydes MR, Keat N, McConnell A, Benson A, Ho A, Roth A, Fortpied C, Eng C, Peckitt C, Coens C, Pettaway C, Arnold D, Hall E, Marshall E, Sclafani F, Hatcher H, Earl H, Ray-Coquard I, Paul J, Blay J-Y, Whelan J, Panageas K, Wheatley K, Harrington K, Licitra L, Billingham L, Hensley M, McCabe M, Patel PM, Carvajal R, Wilson R, Glynne-Jones R, McWilliams R, Leyvraz S, Rao S, Nicholson S, Filiaci V, Negrouk A, Lacombe D, Dupont E, Pauporté I, Welch JJ, Law K, Trimble T, Seymour M (2015). Clinical trial designs for rare diseases: studies developed and discussed by the International Rare Cancers Initiative. Eur J Cancer.

[27] Takken T, Terlingen HC, Helders PJ, Pruijs H, Van der Ent CK, Engelbert RH (2004). Cardiopulmonary fitness and muscle strength in patients with osteogenesis imperfecta type I. J Pediatr.

[28] Tauer JT, Rigo Canevazzi GH, Schiettekatte-Maltais J, Rauch F, Bergeron R, Veilleux L-N (2021). Muscle-bone properties after prolonged voluntary wheel running in a mouse model of dominant severe osteogenesis imperfecta. J Musculoskelet Neuronal Interact.

[29] Gremminger VL, Jeong Y, Cunningham RP, Meers GM, Rector RS, Phillips CL (2019). Compromised exercise capacity and mitochondrial dysfunction in the Osteogenesis Imperfecta Murine (OIM) mouse model. J Bone Miner Res.

[30] Sinkam L, Boraschi-Diaz I, Svensson RB, Kjaer M, Komarova SV, Bergeron R, Rauch F, Veilleux LN (2022). Tendon properties in a mouse model of severe osteogenesis imperfecta. Connect Tissue Res.

